# Female thermal sensitivity and behaviour across the lifespan: A unique journey

**DOI:** 10.1113/EP091454

**Published:** 2024-03-07

**Authors:** Davide Filingeri, Hannah Blount, Alessandro Valenza

**Affiliations:** ^1^ ThermosenseLab, Skin Sensing Research Group, School of Health Sciences The University of Southampton Southampton UK; ^2^ Sport and Exercise Sciences Research Unit, Scienze Psicologiche, Pedagogiche, dell'Esercizio Fisico e della Formazione University of Palermo Palermo Italy

**Keywords:** body temperature regulation, perceptual responses, thermal behaviours, women

## Abstract

Women are a group of individuals that undergo unique anatomical, physiological and hormonal changes across the lifespan. For example, consider the impact of the menstrual cycle, pregnancy and menopause, all of which are accompanied by both short‐ and long‐term effects on female body morphology (e.g., changes in breast size) and temperature regulation, heat tolerance, thermal sensitivity and comfort. However, empirical evidence on how skin thermal and wetness sensitivity might change across the lifespan of women, and the implications that this has for female‐specific thermal behaviours, continues to be lacking. This paper is based on a symposium presentation given at Physiology 2023 in Harrogate, UK. It aims to review new evidence on anatomical and physiological mechanisms underpinning differences in skin thermal and wetness sensitivity amongst women varying in breast size and age, in addition to their role in driving female thermal behaviours. It is hoped that this brief overview will stimulate the development of testable hypotheses to increase our understanding of the behavioural thermal physiology of women across the lifespan and at a time of climate change.

## Abstract (highlights)

- **What is the topic of this review?**
  This review focuses on female thermal sensitivity and behaviour across the lifespan.
- **What is advances does it highlight?**
  Sensing of temperature and wetness are fundamental sensory functions that support heat stress resilience in humans. Recent evidence indicates that women present distinctive thermosensory mechanisms and behaviours that vary as a function of female body morphology and with ageing. Person‐centred thermal innovation should therefore consider the unique and evolving ‘thermosensory needs’ of the female body across the lifespan to help women thrive in a warming climate.

## 1 INTRODUCTION: THE ‘WOMEN AND HEAT’ CHALLENGE

Global warming and the related increase in the frequency and severity of extreme weather events, such as heatwaves, is now the greatest threat to human prosperity and survival (IPCC, 2023). A recent analysis of the heat‐related mortality during Europe's 2022 summer heatwave estimated 61,672 heat‐related deaths (Ballester et al., 2023). Notably, the burden of heat‐related mortality during Europe's 2022 summer heatwave was higher amongst women (i.e., 56% more heat‐related deaths in women than in men relative to population), which was probably attributable to differences with men in physiological, age structure and sociocultural factors (Ballester et al., 2023). Beside their impact on mortality, hot weather and heat extremes severely limit the work and exercise capacity of people, with consequent detrimental effects on the health, comfort and productivity of individuals (Ebi et al., 2021).

Thermal behaviour [i.e., the ability to detect the thermal state of one's own body and surroundings and actively to pursue thermal comfort (Schlader & Vargas, 2019)] represents the most effective mechanism to maintain thermal homeostasis and ensure heat stress resilience (Vargas et al., 2023). The efficacy of this adaptive behaviour is reliant on the ability to detect variations in our internal (i.e., body) and external environment by sensing changes in temperature and wetness (Vargas et al., 2020a). Our understanding of the molecular and neurophysiological mechanisms underpinning human perception of temperature and wetness has expanded significantly in the last 30 years (Filingeri, 2016), but we lack comprehensive evidence on the complex interplay amongst autonomic, perceptual and behavioural responses to heat and on their individual variability, for example as a function of sex, age and hormonal status (Greenfield et al., 2023; Lei et al., 2019).

Women are a group of individuals that undergo unique anatomical, physiological and hormonal changes across the lifespan. For example, consider the impact of the menstrual cycle, pregnancy and menopause, all of which are accompanied by both short‐ and long‐term effects on female body morphology (e.g., changes in breast size) (Wade et al., 2010) and temperature regulation, heat tolerance, thermal sensitivity and comfort (Carter et al., 2023; Lei et al., 2019; Smallcombe et al., 2021; Yanovich et al., 2020). Surprisingly, women continue to represent a very small proportion of participants tested in thermoregulation research (e.g., 12%–18% of the total over the last 10 years) (Hutchins et al., 2021).

Empirical evidence indicates that innate differences in skin thermal and wetness sensitivity might exist between men and women and that this could underlie their divergent behavioural responses to heat stress (Greenfield et al., 2023). However, knowledge on how thermal and wetness sensitivity might differ amongst women varying in body morphology and age, and the implications that this might have for female‐specific thermal behaviours during heat stress, continue to be lacking. This knowledge gap provides a significant barrier to the development of interventions (e.g., personalized cooling) and solutions (e.g., sport garments and thermal wearables) that meet the unique thermal needs of women across different life stages and which, ultimately, facilitate the maintenance of an active lifestyle on a warming planet.

**FIGURE 1:**
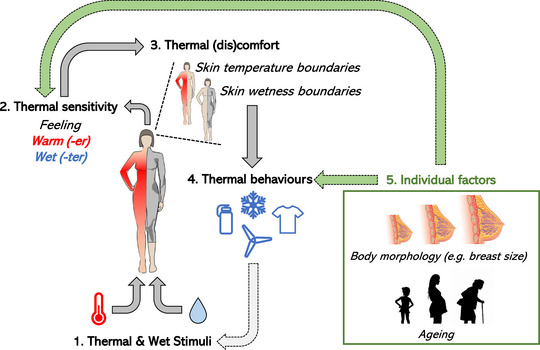
A conceptual framework for female thermal sensitivity and behaviour. Changes in body temperature and skin wetness (1) trigger conscious perceptions of temperature and wetness (2). These ‘ongoing’ perceptions are compared with the ranges of skin temperature and wetness that are deemed comfortable (3). If skin temperature and wetness boundaries are exceeded by ongoing perceptions (e.g., feeling too warm and wet), thermal discomfort is experienced (3) and thermal behaviours are triggered [(4) e.g., physical cooling, hydration, changing clothing insulation)] to restore the body's thermal state and comfort. Emerging evidence on female‐specific individual factors (5) indicates that body morphology and ageing could modify either an individual's thermal sensitivity (i.e., influence on 2) or their behavioural responses to the same thermal stimulus (i.e., influence on 4).

This paper is based on a symposium presentation given at Physiology 2023 in Harrogate, UK. It aims to review new evidence on anatomical and physiological mechanisms underpinning changes in skin thermal and wetness sensitivity in women varying in breast size and age (i.e., older vs. younger adults) and on their role in driving female thermal behaviours (Figure 1). Besides being a current focus of our laboratory's research programme on women's thermal sensitivity, understanding the role of breast size and ageing on women's thermal sensitivity could offer exemplars of both the technological (e.g., to innovate the design of sport bras) and public health impact (e.g., to inform heat protection measures in the most vulnerable) of such women‐centric investigations.

## 2 FEMALE BODY MORPHOLOGY AND BREAST THERMAL SENSITIVITY

Whether perceptual sensitivity to increases or decreases in skin temperature (hereby referred to as thermal sensitivity) differs between men and women is a topic that continues to be debated widely (Greenfield et al., 2023; Kingma & van Marken Lichtenbelt, 2015), owing to its broad applications in the design of (sport) clothing and of thermal comfort systems in the building and automotive industries (Luo et al., 2020). Detailed consideration of this topic is beyond the scope of this review, and the interested reader is referred to the excellent review by Greenfield and colleqgues (Greenfield et al. 2023). Nevertheless, recent empirical evidence fuelling the aforementioned debate and relating to the mechanisms of spatial summation in the thermal sense (whereby thermal sensations change depending on the size of the stimulated area; see e.g., Filingeri et al., 2018; Luo et al., 2020) has offered a new framework to investigate within‐sex differences in thermal sensitivity in women varying in body morphology, specifically in breast size and surface area.

Let us first consider the mechanisms of spatial summation. We have long known that, given the same local temperature stimulation of the skin, the resulting thermal sensation can vary in magnitude depending on the size of the stimulated area (i.e., the larger the area, the more intense the resulting hot or cold sensation) (Hardy & Oppel, 1937; Stevens et al., 1974). This phenomenon, known as spatial summation, might reflect a change in the precision of the neural representation of a change in skin temperature, which improves the ability of the nervous system to distinguish true thermal stimuli from sensory noise (Courtin et al., 2023). This mechanism has recently gained attention in the context of better understanding differences in thermal sensitivity commonly reported to exist between men and women (Greenfield et al., 2023). Indeed, when considering evidence from studies performing thermal sensitivity mapping (see, e.g., Filingeri et al., 2018; Gerrett et al., 2014; Luo et al., 2020), it appears that the greater thermal sensitivity of women than men might result from their smaller (on average) body surface area, which, in turn, leads to a greater proportion of their body being thermally stimulated by the same fixed‐size thermal stimulus (Luo et al., 2020). Importantly, this evidence has highlighted a body morphology‐dependent mechanism that might be implicated in individual differences in thermal sensitivity (Greenfield et al., 2023).

The female body is unique in that it undergoes significant morphological changes across specific body parts, such as the breast. In particular, female breast development and the resulting breast surface area can vary greatly owing to genetic factors, body mass index and energy intake early in life (Wade et al., 2010). This sex‐specific anatomical feature offers the opportunity to test the body morphology‐dependent mechanism by evaluating whether breast thermal sensitivity varies amongst women differing in breast size and surface area. Our group has recently tackled this question in a cohort of 22 young women (∼28 years of age) of varying breast sizes (i.e., from small to extra‐large; breast surface area range, 147–561 cm^2^). Our preliminary unpublished observations indicated that local thermal sensitivity of the breast is constant across breast sizes, meaning that increases in breast size are not accompanied by a decrease in local thermal sensitivity, as we had originally hypothesized. Interestingly, the same cohort of young women presented sweat gland densities and local sweat rates at the breast that decreased linearly with increasing breast size. The latter observation is likely to be dependent on the fact that the maximum expression of sweat glands across the skin (i.e., 2–5 million) is achieved before (i.e., ∼2 years of age) female breast development (Kuno, 1956); as a result, skin expansion during breast growth is likely to decrease sweat gland density. We had expected that breast growth might also have decreased the density of thermoreceptors innervating larger breasts, with consequent reductions in local breast sensitivity. However, such a response was not observed in our cohort of women. This observation provides psychophysical evidence in support of the intriguing hypothesis that breast growth might be accompanied by an enlargement of the receptive fields associated with those thermoreceptors that present multiple temperature‐sensitive spots on the skin (Kenshalo & Gallegos, 1967). Whether such changes in receptive field size also apply to scenarios in which rapid weight gain or loss occurs or during pregnancy remains unclear; however, their investigation could provide relevant empirical evidence for such a receptive‐field model.

From a fundamental standpoint, these studies provide new, female‐specific physiological evidence that perceptual (i.e., thermal sensitivity) and autonomic (i.e., sudomotor) mechanisms involved in thermal regulation and behaviours might be affected differently by sex‐specific changes in body morphology across unique body parts, such as the female breast. Increasing our fundamental understanding of women‐centric thermal perception and sweating mechanisms at the breast has important applied implications, because it could inform the design of wearables and accessories, such as sport bras, that meet the thermal and comfort needs of women varying in breast size. This could, in turn, facilitate the maintenance of an active lifestyle across the lifespan, with related health benefits for the global female population (Valenza et al., 2023b).

## 3 EFFECTS OF AGEING ON THERMAL BEHAVIOUR

The physiological mechanisms underlying human thermal behaviours during heat stress have been the subject of intense investigations over the past 50 years [see e.g., classic work by Michelle Cabanac (Cabanac, 1971, 2011) and more recent work by Schlader and colleagues (Schlader & Vargas, 2019; Schlader et al., 2010)], including a recent focus on how individual characteristics, such as sex or clinical status, can modulate these adaptive behaviours (Christogianni et al., 2023; Vargas et al., 2019, 2020b). Nevertheless, female‐specific investigations on behavioural responses to thermal stress across the lifespan remain limited, and they have started to emerge only very recently [i.e., see research performed by Carter and colleagues (Carter et al. 2023) and by our group (Valenza et al., 2023a)].

Although the recent work by Carter et al. (2023) did not assess changes in thermal behaviour per se, this research has provided new evidence that older (menopausal) women (∼55 years of age) presented a lower tolerance to increasing air temperatures when compared with younger (non‐menopausal) counterparts (∼46 years of age). Given that the (un)acceptability of environmental conditions (e.g., heat) is likely to drive thermal behaviours (e.g., seeking active cooling), it would have been reasonable to hypothesize that, if given the opportunity, the cohort of older women tested by Carter et al. (2023) might have engaged in earlier thermal behaviours during heat stress than the younger cohort.

This hypothesis has been tested recently by our group in a combined study, in which younger (∼25 years of age) and older (∼53 years of age) women were assessed both for their regional skin sensitivity to thermal and wet stimuli and for their cool‐seeking thermal behaviour during increases in body temperature elicited by exercise (Valenza et al., 2023a). The key takeaway of our study is that older women presented both a reduction in their autonomic heat‐defence responses (i.e., changes in mean skin temperature, physical skin wetness and whole‐body sweat losses) and a shift in their reliance from mostly central (i.e., changes in core temperature) to more integrated central and peripheral thermo‐afferent signals (i.e., core temperature, physical skin wetness and temperature) to drive cool‐seeking behaviours (Valenza et al., 2023a). Our study also indicated that body regional patterns of thermal and wetness sensitivity did not decline in older women to the extent that we have recently observed in older men (∼58 years of age) (Wildgoose et al., 2021). This latter finding is intriguing, because it opens the as yet untested question of whether the shift by older women towards a greater reliance on (mostly preserved) peripheral thermosensory inputs to drive cool‐seeking behaviour might offer additional (behavioural) protection during heat stress, in comparison to the (likely) responses of older, less thermally sensitive, men.

A better understanding of the complex interplay between ageing and biological sex in behavioural thermoregulation has important implications for development of more holistic models of the individual risk of heat vulnerability (Vargas et al., 2023), which can then inform heat‐protection planning to mitigate the health impact of extreme heat and humidity in vulnerable groups, such as older women (Baldwin et al., 2023; Ballester et al., 2023; Vanos et al., 2023, 2020).

## 4 CONCLUSIONS

Women undergo a unique thermophysiological journey across their lifespan. Further physiological and applied knowledge is needed to gain a better understanding of the responses of women to thermal stressors, to develop more women‐centric approaches to thermal health, safety and comfort. We hope that this brief overview on emerging female‐specific mechanisms of thermosensory function will stimulate the development of testable hypotheses to increase our understanding of the behavioural thermal physiology of women across the lifespan at a time of climate change.

## AUTHOR CONTRIBUTIONS

Davide Filingeri conceived and drafted the work. Hannah Blount and Alessandro Valenza acquired, analysed and interpreted data for the work. All authors approved the final version of the manuscript and agree to be accountable for all aspects of the work in ensuring that questions related to the accuracy or integrity of any part of the work are appropriately investigated and resolved. All persons designated as authors qualify for authorship, and all those who qualify for authorship are listed.

## CONFLICT OF INTEREST

No competing interests declared.
